# How to improve prescription of inhaled salbutamol by providing standardised feedback on administration: a controlled intervention pilot study with follow-up

**DOI:** 10.1186/s12913-015-0702-x

**Published:** 2015-01-28

**Authors:** Martina P Neininger, Almuth Kaune, Astrid Bertsche, Jessica Rink, Juliane Musiol, Roberto Frontini, Freerk Prenzel, Wieland Kiess, Thilo Bertsche

**Affiliations:** Drug Safety Center, University of Leipzig, Leipzig, Germany; Department of Clinical Pharmacy, University of Leipzig, Leipzig, Germany; Department of Women and Child Health, Hospital for Children and Adolescents and Center for Pediatric Research, University of Leipzig, Leipzig, Germany; Pharmacy Department of the University Hospital Leipzig, University of Leipzig, Leipzig, Germany

**Keywords:** Prescription, Administration, Salbutamol, Paediatric, Dosing, Inhalation

## Abstract

**Background:**

The effectiveness of inhaled salbutamol in routine care depends particularly on prescribed dosage and applied inhalation technique. To achieve maximum effectiveness and to prevent drug-related problems, prescription and administration need to work in concert.

**Methods:**

We performed a controlled intervention pilot study with 4 consecutive groups in a general paediatric unit and assessed problems in salbutamol prescribing and administration. Control group [i]: Routine care without additional support. First intervention group [ii]: We carried out a teaching session for nurses aimed at preventing problems in inhalation technique. Independently from this, a pharmacist counselled physicians on problems in salbutamol prescribing. Second intervention group [iii]: Additionally to the first intervention, physicians received standardised feedback on the inhalation technique. Follow-up group [iv]: Subsequently, without any delay after the second intervention group had been completed, sustainability of the measures was assessed. We performed the chi-square test to calculate the level of significance with p ≤ 0.05 to indicate a statistically significant difference for the primary outcome. As we performed multiple testing, an adjusted p ≤ 0.01 according to Bonferroni correction was considered as significant.

**Results:**

We included a total of 225 patients. By counselling the physicians, we reduced the number of patients with problems from 55% to 43% (control [i] vs. first intervention [ii], n.s.). With additional feedback to physicians, this number was further reduced to 25% ([i] vs. [iii], p < 0.001). In the follow-up [iv], the number rose again to 48% (p < 0.01 compared to feedback group).

**Conclusions:**

Teaching nurses, counselling physicians, and providing feedback on the quality of inhalation technique effectively reduced problems in salbutamol treatment. However, for success to be sustained, continuous support needs to be provided.

**Trial registration:**

German Clinical Trials register: DRKS00006792.

## Background

Children are prone to preventable adverse drug events caused by drug-related problems (DRP). The rate of those events in this patient group is three times higher than in adults and 79% of potential adverse drug events occur in prescribing [1]. Although these results have been reported, data on the actual rate of prescribing errors in paediatrics is scarce [2]. Ghaleb et al. found prescribing error rates of up to 31% in children [3]. One of the main problems in paediatric prescribing is patient-individualized dosing. Published dosage recommendations for children, however, are very heterogeneous [4]. This applies especially to salbutamol which rates amongst the most frequently prescribed drugs in children [5,6]. The optimal dosage of salbutamol in children is contested [7,8] and imprecise dosing instructions may lead to DRP. Additionally, the medication’s effectiveness does not only depend on the prescribed dosage but also on the patient’s inhalation technique [9]. Various methods such as guideline implementation, teaching programmes, ward round services by pharmacists, and electronic prescribing are described to solve DRP [10-14]. Existing studies focus either on prescription or administration. Several studies dealing separately with both processes exist. To the best of our knowledge, however, no studies on the effects of intervention strategies addressing the interrelation between prescription and administration have been published. Therefore, we performed a controlled interventional study with 4 consecutive groups: [i] control (no additional support, routine care), [ii] first intervention (implementation of an internal guideline, training sessions for nurses, pharmaceutical counselling), [iii] second intervention (additional feedback to the prescribing physicians on the quality of the inhalation processes) and [iv] follow-up (termination of all additional support, routine care).

Thus, we linked prescription and administration by a standardised flow of information to the prescribing physicians and investigated its benefit.

## Methods

### Setting

This pilot study was performed in a general paediatric unit with 22 beds of a university hospital. The unit focused among others on pulmonary diseases. Computerised physician order entry, a clinical decision support system, unit dose, or other pharmaceutical services were not yet implemented in this unit.

### Inclusion and exclusion criteria

We included all consecutive patients with salbutamol prescriptions who had been admitted to the participating unit due to bronchial obstruction or asthma. No additional examinations/laboratory data were required for inclusion. Patients with a hospital stay restricted to one weekend were excluded because the pharmaceutical counselling was offered on weekdays only.

### Definitions

#### Drug-related problem (DRP)

DRP refers to deviations from an internal guideline on bronchial obstruction or inadequate documentation such as incomplete prescriptions (e.g. missing single dose or frequency), incomprehensible or ambiguous prescriptions, or not clearly marked discontinued medications.

#### Guideline

An internal guideline addressed appropriate drug prescription and dosing in children suffering from obstructive lung diseases and asthma including standardised salbutamol treatment. This guideline was developed by an interdisciplinary expert panel and was put into practice at the beginning of the first intervention group of this study. The guideline was based on the national guideline on the treatment of asthma [15]. Furthermore, our guideline included *two dose regimens*: a *regular* salbutamol dose treatment regimen and a *high* dose regimen for children suffering from severe obstruction or with insufficient inhalation technique (Table 1). Doses exceeding this high dose regime were defined as *elevated* doses.

**Table 1:** **Excerpt of the internal guideline: recommendations on treatment of bronchial obstruction**

	**Inhaler device**	**Children <12 years**	**Children >12 years**	**Interval (minimum)**
**Regular dosage regimen**	**Nebulization (1 drop = 0,25 mg)**	1-2 drops per life year, min. single dose 3 drops (recommendation by the local Pediatric Pneumology Section), max. single dose 8 drops; 3-4 times/day	5-10 drops, 3-5 times/day, max. daily dose 50 drops	4 hours
	**Metered dose inhaler (MDI) (1 puff = 100 μg)**	1 puff 3-4 times/day; as needed up to 6 puffs/day	1-2 puffs 3-4 times/day; as needed up to 12 puffs/day	3 hours
**High dosage regimen**	• Recommendation in cases of severe obstruction or insufficient inhalation technique according to the local Pediatric Pneumology Section: 2 puffs 6 times/day (MDI)			
	• Note: inform family/carer on off-label use; higher doses need to be discussed with a senior physician			
**General remarks**	• Children <5 years: prefer MDI with spacer (use facemask if necessary)			
	• Increased single dose/frequency possible as needed for symptoms in individual cases (off-label)			
	• Good response to salbutamol/recovery from symptoms: reduce interval between inhalations to 3 hours (MDI) or 4 hours (nebulization)			
	• No oral β_2_-agonists in acute situations			
**Disclaimer**	• The recommendations cover only routine care situations, in individual cases therapy has to be adjusted to the individual needs.			
	• The therapy decision is the sole responsibility of the prescribing physician.			
	• This information does not replace the approved summary of product characteristics.			

Legend: if not stated otherwise: dosage approved by the national authority.

#### Expert panel

The expert panel was composed of three pharmacists and four paediatricians, among them the head of the local Pediatric Pneumology Section. The panel was in charge of developing the internal guideline.

#### Monitoring of inhalation technique

Two pharmacists monitored and analysed the quality of patients’ inhalation processes. Patient-individual parameters such as breathing coordination, cooperation during inhalation process, and body position were rated. We also assessed device specific administration rules such as sufficient number of breaths in case of using a spacer, air-tight fit of mask/mouthpiece, and proper shaking of metered dose inhaler (MDI).

#### Teaching session (for nurses)

A teaching session for nurses was developed and implemented to reach “best practice” in inhalation technique. We offered a 60-minute teaching session, in which a pharmacist explained inhalation handling guidelines and provided background information on the impact of the inhalation technique on clinical outcomes. The teaching session focused on the correct use of nebuliser and MDI with and without spacer as well as on the question of how to conduct an optimal inhalation maneuver with children. We offered the teaching session five times in order to give as many nurses as possible the opportunity to take the session. Furthermore, nurses had the opportunity to ask the pharmacists for advice during the monitoring. All nurses working in the unit during the study were invited to take part in the study.

#### Counselling service (for physicians)

A pharmacist assessed salbutamol prescriptions each day and advised prescribing physicians as regards DRP and possibilities to solve them during the next morning ward round. The pharmacist’s recommendation was patient-individualised in respect to the patient’s physical and clinical condition. This strategy focused on the appropriateness of prescribed dosing.

#### Feedback service (for physicians)

In addition to providing a counselling service, pharmacists reported to the physicians the quality of inhalation technique assessed by monitoring. To standardise this feedback a structured reporting form on the monitored items was filed into the patient’s chart. This information on patient’s personal inhalation abilities in addition to the patient’s physical and clinical condition allows patient-individual recommendations on salbutamol prescribing.

### Study protocol

Following approval by the Ethics Committee of the University of Leipzig, Leipzig, Germany, and the responsible employee committee, we performed a prospective controlled interventional pilot study focusing on salbutamol prescribing. Primary outcome was the number of patients with at least one predefined DRP in their salbutamol prescribing.

The study consisted of 4 consecutive groups: [i] *control group* (no additional support, routine care), [ii] *counselling group* (with teaching the nurses, implementation of the internal guideline and counselling service for the physicians, additionally to routine care), [iii] *feedback group* (with monitoring of the nurses, counselling and feedback service for the physicians, additionally to routine care) and [iv] a *follow-up group* (any additional support was terminated, routine care only). The follow-up was performed subsequently without any delay after the feedback group had been completed.

An additional teaching session was offered at the beginning of the counselling group to inform all physicians about the aim of the study and to explain the instruments of the study programme, especially the newly developed guideline. Furthermore, the guideline was placed at the medical trolley in the unit and sent by email to the concerned physicians. The counselling service and data collection was launched in the week after the teaching session.

### Power calculation and data analysis

Considering similar studies of our group [16] and our experience in this study setting, we assumed that in around 55% of the patients at least one DRP would occur. A relative reduction of at least 50% after the full intervention programme (counselling and feedback service) was considered clinically relevant (i.e. a rate of ≤27.5% in the feedback group, primary outcome). Assuming rates in this range, a two-sided chi-square test at significance level of α = 0.05 and sample size of 41 per group would provide a power of 1 − β = 0.80 for the primary endpoint (calculated by G*Power Version 3.1.7, Franz Faul, University of Kiel, Germany [17]).

Data is presented as median with first (25%) and third (75%) quartile (Q25/Q75) and minimum/maximum as appropriate. We performed the Kruskal-Wallis-test or chi-square test as appropriate with Kyplot® (KyensLab, Tokyo, Japan) to calculate the level of significance with p ≤ 0.05 considered to indicate a statistically significant difference for the primary outcome. As we performed multiple testing, we applied the Bonferroni correction and considered an adjusted p ≤ 0.01 as significant. Inter-rater agreement was calculated with Cohen's kappa. Kappa values (κ) of 0.81-1.00 represent very good agreement between two different monitors.

## Results

We included patients as shown in Table 2.

**Table 2:** **Characteristics of included patients with salbutamol treatment of bronchial obstruction or asthma**

	**Control group**	**Counselling group**	**Feedback group**	**Follow-up group**
Total number of patients	56	56	57	56
Among them female	22 (39%)	21 (38%)	18 (32%)	19 (34%)
Median age in years	1.27	1.21	0.67	1.47
Q25/75	0.41/3.55	0.35/2.40	0.34/1.88	0.62/4.56
Minimum/maximum	0.30/14.03	0.06/14.68	0.10/13.86	0.03/17.81

Legend: data is presented as median with first (25%) and third (75%) quartile (Q25/Q75); there are no differences in sex or age between the four groups (p-values not significant).

**Figure 1:**
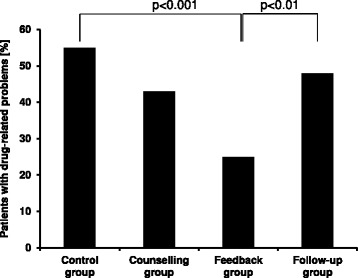
**Patients with drug-related problems (DRP) in prescription in their salbutamol treatment.** DRP refers to deviations from an internal guideline or inadequate documentation [incomplete prescriptions (e.g. missing single dose or frequency), incomprehensible or ambiguous prescriptions, not clearly marked discontinued medications]. n (Control group) = 56, n (Counselling group) = 56, n (Feedback group) = 57, n (Follow-up group) = 56.

By counselling the prescribing physicians [ii], we reduced the number of patients with DRP from 55% (31 of 56 patients, control group [i]) to 43% (24/56, n.s.). Additional feedback [iii] led to a further decrease in the number of patients with DRP as the primary outcome to 25% (14/57; p < 0.001 compared to control group [i], Figure 1). After termination of any additional support, the number of patients with DRP increased again to 48% (27/56) in the follow-up [iv] (p < 0.01 compared to feedback group [iii]).

As main reason for DRP in prescription we identified elevated salbutamol doses related to the internal guideline. The number of patients with elevated doses was 50% (28/56) in the control group [i], 29% (16/56) in the counselling group [ii], 18% (10/57) in the feedback group [iii], and 34% (19/56) in the follow-up group [iv] (Table 3).

**Table 3:** **Dosing and drug-related problems in prescription**

**Categories** ^**a**^	**Control group**	**Counselling group**	**Feedback group**	**Follow-up group**
	**Patients n (%)**	**Patients n (%)**	**Patients n (%)**	**Patients n (%)**
According to guideline				
Regular dose regimen^b^	17 (30)	17 (30)	24 (42)	9 (16)
High dose regimen^b^	8 (14)	15 (27)	19 (33)	20 (36)
Deviating from guideline^c^				
Elevated doses^b^	28 (50)	16 (29)	10 (18)	19 (34)
Inadequate documentation	12 (21)	11 (20)	4 (7)	17 (30)

^a^Multiple categories possible; ^b^The guideline included *two dose regimens*: a *regular* salbutamol dose treatment regimen and a *high* dose regimen for children suffering from severe obstruction or with insufficient inhalation technique. Doses exceeding this high dose regime were defined as *elevated* doses; ^c^we did not identify any cases of underdosing.

We monitored a total of 255 inhalation processes, 156 with a MDI and 99 with a nebulizer. The feedback given to the physicians is shown in Table 4. For example, proper shaking of the MDI was reported in 90% of the monitored inhalation processes.

**Table 4:** **Positive feedback to the prescribing physicians on the quality of the inhalation processes in the feedback group**

**Item**	**All processes (MDI + nebulizer)**	**MDI**	**Nebulizer**
**Total number of processes**	255	156	99
**Device specific administration rules**			
Good airtight fit of mask/mouthpiece	202 (79%)	137 (88%)	65 (67%)
MDI^a^ shaken before administration	-	141 (90%)	-
Sufficient number of breaths when using a spacer	-	113 (72%)	-
**Patient-individual parameters**			
Good upright body position	101 (40%)	67 (43%)	34 (34%)
Good cooperation^b^	148 (58%)	77 (49%)	71 (72%)
Good breathing coordination	19 (7%)	13 (8%)	6 (6%)

^a^MDI: Metered Dose Inhaler ^b^Good cooperation was defined as patient’s support of the administration activities with regard to the age-dependent abilities of the patient, i.e. for younger children no rejection of the administration process or correctly attending to the nurses’ instructions for older children and adolescents.

For each monitored inhalation process a reporting form with the assessment of each of the listed items was filed into the patient’s chart; positive feedback was forwarded if the administration processes were appropriate in the respective item and a negative feedback in cases if administration was inappropriate.

Inter-rater agreement for the two independent monitors was very good with κ = 0.96.

## Discussion

Prescription and administration of inhaled drugs cause major problems in the treatment of obstructive lung diseases [18,19]. Children are of special interest because they are frequently affected by obstructive lung diseases requiring appropriate treatment. Age-dependent dosing is not yet sufficiently investigated by randomized controlled trials. Therefore, in routine practice off-label use with doses higher than approved without sufficient evidence for better outcomes is frequent [20]. Particularly inhaled drugs such as salbutamol require additional consideration of the administration technique to decide on the appropriate dosage. We identified an alarming percentage of patients with DRP in salbutamol prescriptions in routine care [i]. Implementation of a guideline and a counselling service addressing salbutamol prescription performed by a pharmacist decreased the high rate of DRP [ii]. The breakthrough, however, was achieved by providing additional feedback to physicians addressing the inhalation technique of the patient assessed by a monitoring [iii]. By implementing a programme that included also a teaching session for nurses to improve inhalation technique, a guideline as standard for salbutamol prescribing and standardised feedback on the inhalation technique, we particularly prevented the prescription of elevated doses. The pay-off of our programme decreased in the follow-up and was no longer detectable after the intervention had been terminated [iv].

Patient outcomes in obstructive lung diseases in routine care are not as good as in clinical trials due to poor inhalation technique [19]. Poor inhalation technique results inter alia from problems in handling the inhalation devices or knowledge deficits. Therefore, patients need special guidance in the inhalation process by healthcare professionals. However, correct inhaler use was demonstrated by only 15-69% of healthcare professionals [19]. Their knowledge on correctly using an inhalation device was also insufficient [21]. Comprehensive education on inhalation technique decreased deficits in inhalation technique resulting in improved clinical parameters at even lower doses than originally applied [22]. To address the relation between the prescribed dosage and administration, we instructed nurses on how to optimise inhalation technique and, simultaneously, we implemented a guideline on the treatment of obstructive lung diseases for physicians. This is essential, because the dose finally reaching the patient might be too low to be effective, if prescribed dosages are reduced without an improvement of inhalation technique. Otherwise, if inhalation processes are optimised without adopting the prescribed salbutamol dose, patients may suffer from increased adverse drug effects due to elevated salbutamol exposure. Therefore, both processes should be tuned to each other especially if they undergo changes.

In salbutamol inhalation therapy, the optimal dosage depends on the inhalation technique, the clinical condition of the patient and the dosage regimen. In the counselling group, detailed information on the inhalation technique was not yet available to the physicians. Thus, daily doses were reduced only to a minor degree. In the feedback group, the (improved) quality of inhalation technique in the individual patient was reported to the physicians resulting in reduced prescribed salbutamol doses. Especially the number of patients with salbutamol doses higher than recommended in the guideline was effectively reduced. After the full programme was implemented, the number of patients treated with the high dose regimen doubled indicating that those children were now sufficiently treated with high doses if necessary, but they were no longer prescribed too much treatment. The high number of items reported as “good” in the feedback indicates a successful training. Due to the very young age of the patients, breathing coordination remains problematic because young children do not have the abilities to support the inhalation process yet.

The benefit of pharmacists in the paediatric setting has also been discussed by Fortescue et al.: a high degree of all medication errors in prescribing, transcribing and administering could be prevented by pharmacists [23]. Furthermore, improved communication between physicians, nurses, and pharmacists was suggested as potentially effective intervention to prevent medication errors in the same study. We successfully developed and performed an interventional pilot study on evaluating a comprehensive programme to optimise the information process by offering standardised feedback on the inhalation technique to the physicians. As a consequence, the rate of preventable DRP decreased. A mere implementation of a guideline without any further support in prescription was insufficient as the number of patients affected by DRP rose again to base level after all additional support had been terminated.

### Limitations

We assessed DRP in children as a high risk group for DRP in prescription (off-label use and missing data from randomised clinical trials) as well as in administration (children have no or limited abilities to support inhalation actively). Therefore, the results are not transferable to other patient populations. In our setting, no routine assessment of safety and effectiveness is documented by the prescribing physicians. Our intention, however, was an assessment under routine conditions. We therefore did not implement any additional assessment instruments. What is more, the implementation of those additional instruments could have influenced the outcomes themselves. DRP are correlated with the occurrence of adverse drug reactions and missing effectiveness [13]. Therefore, strategies should intercept DRP to prevent adverse drug reactions before they actually occur. By assessing DRP instead of occurring adverse drug reactions we intended to evaluate the “first” parameter on the cascade to identify potential risks for the patients. Additionally, this parameter can be identified under routine conditions. Effectiveness and safety including recurrence or rehospitalisation, however, are sometimes difficult to be correlated to the actual use of a drug and requires high numbers of patients to reach significant levels.

We did not choose a blinded and randomized procedure. It is difficult to perform blinded teaching interventions or to randomize patients for interventions influencing the knowledge and practical performance of health care professionals treating control and intervention patients at the same time. Therefore, we considered a cluster randomization for our study including similar units in different centres in our city. However, we found in a pre-testing that the settings were quite different. We did not perform a multicentre study in different cities with similar units because of the limited resources of this study.

## Conclusions

We identified a high number of DRP in inhaled salbutamol therapy in routine patient care. By providing a counselling service at the point of prescription based on a newly developed and implemented guideline DRP rates and elevated doses in salbutamol prescription decreased. Those effects were strongly enhanced by providing feedback which passed on information on the inhalation technique to the prescribing physician: we halved the number of patients affected by DRP compared to control. To stabilize those outcomes, however, continuous prescription review and counselling as well as reporting on inhalation technique were shown to be necessary to achieve sustainable effects in routine care.

## Abbreviations

DRP: Drug-related problem
MDI: Metered dose inhaler
